# Entropy Production in Electroosmotic Cilia Facilitated Stream of Thermally Radiated Nanofluid with Ohmic Heating

**DOI:** 10.3390/mi12091004

**Published:** 2021-08-24

**Authors:** Najma Saleem, Sufian Munawar, Ahmer Mehmood, Ibtisam Daqqa

**Affiliations:** 1 Department of Mathematics and Natural Sciences, Prince Mohammad Bin Fahd University, Khobar 31952, Saudi Arabia; idaqqa@pmu.edu.sa; 2 Department of Quantitative Methods, College of Business Administration, Imam Abdulrahman Bin Faisal University, P.O. Box 1982, Dammam 34212, Saudi Arabia; smunawar@iau.edu.sa; 3 Department of Mathematics and Statistics, FBAS, International Islamic University, Islamabad 44000, Pakistan; ahmerqau@yahoo.co.uk

**Keywords:** entropy analysis, electroosmotic ciliary flow, thermal radiation, magnetic field, Joule heating, Carreau nanofluid

## Abstract

No thermal process, even the biological systems, can escape from the long arms of the second law. All living things preserve entropy since they obtain energy from the nutrition they consume and gain order by producing disorder. The entropy generation in a biological and thermally isolated system is the main subject of current investigation. The aim is to examine the entropy generation during the convective transport of a ciliated nano-liquid in a micro-channel under the effect of a uniform magnetic field. Joint effects of electroosmosis and thermal radiation are also brought into consideration. To attain mathematical simplicity, the governing equations are transformed to wave frame where the inertial parts of the transport equations are dropped with the use of a long-wavelength approximation. This finally produces the governing equations in the form of ordinary differential equations which are solved numerically by a shooting technique. The analysis reports that the cilia motion contributes to enhance the flow and heat transfer phenomena. An enhancement in the flow is observed near the channel surface for higher cilia length and for smaller values of the electroosmotic parameter. The entropy generation in the ciliated channel is observed to be lessened by intensifying the thermal radiation and decreasing the Ohmic heating. The extended and flexible cilia structure contributes to augment the volumetric flow rate and to drop the total entropy generation in the channel.

## 1. Introduction

Motile cilia aided transport plays an important role in the motion of the cell body or the neighboring material over the cell surface. Cilia consists of minuscule hair resembling threads that move periodically to move in a bio-fluid. They move similar to sculls, lashing backward and forth, collide in synchronization, and generate a pattern of coordinated traveling waves along the wall called metachronal waves. Thus, an escalation in the liquid stream is produced due to the force exerted on the fluid through power strokes. Motile cilia have varied usage in the disciplines of physiology and bioengineering. For instance, in the respiratory system [1] cilia are accountable for clearing the airways by eradicating mucus and dust particles, cilia contribute to drive the food to its ultimate end [2] in the digestive tract, to transport eggs via oviducts in female fallopian tubes [3,4], and to ductile afferents mix sperms in the male testis to control them from congregating and blocking the tubules [5].

Artificial cilia-structured micro-scale electromechanical devices such as sensors, actuators, and lab-on-a-chip devices have nowadays gained significant interest from scientists. These microfluidic tools, triggered by electric and magnetic forces, have been widely used in hemodialysis, drug supply, and micromixers in nano and microfluidic pumps, etc. To emphasize the significance of ciliary motion in biology and bioengineering fields, some important studies have been mentioned for the interested audience [6,7,8].

An electroosmotic motion evolves as a result of the submission of the electric field on the liquid. This flow is associated with an electric double layer (preserving the net charge density) that develops at the solid-fluid borderline. Examples of some advanced electrical machines in micro and nanofluidic devices are electroosmotic liquid pumps, DNA testing, pharmaceutical drug supply pump, cooling chips, lab-on-a-chip devices, and microfabricated liquid devices, etc. In this regard, the pioneering contribution was made in 1964 by Burgreen and Nakache [9]. Abo-Elkhair et al. [10] considered an electric double layer and partial slip in a peristaltic motion and established that the high values of an electroosmotic parameter hinder the fluid motion near microchannel walls. Chaube et al. [11] analyzed the impact of electric field on non-Newtonian liquid and emphasized the importance of this study in lab-on-chip devices and micropumps. A theoretical analysis dealing with an axially applied electric field on the peristaltic motion of Jeffrey fluid was delivered by Ramesh et al. [12]. Javayel et al. [13] considered the electroosmotic peristaltic motion of nanofluid and determined that the skin friction at the pump walls increases for the elevated values of an electroosmotic parameter. Hang et al. [14] studied the electroosmosis-driven stream in a microchannel with a stretching upper boundary. Some current studies with the concept of combined electric and magnetic forces in a peristaltic flow through a microchannel are reported in [15,16,17].

One of the major issues in the preparation of energy-resonant materials is the low thermal conductance of traditional heat transfer fluids. Nowadays, this issue is being addressed by suspending nanoscale solid granules such as copper, silver, gold, titanium, copper oxide, etc., in a conventional liquid such as water, ethylene glycol, oil, or blood, etc. This homogeneous mixture is called nanofluid which has efficient thermal performance and serves as an optimal heat transfer medium. Novel applications of nano-liquids are seen in fuel batteries, thermal spread in micro-electrical devices, refrigerators, engine cooling, chiller, healing, and therapeutic procedures. Various biomedical applications, specifically drug delivery, involve incorporating the Copper-nanoparticles or Gold-nanoparticles in blood transport for therapeutic effectiveness. An interesting study by Majewski et al. [18] shows how Cu-nanoparticles increase the antioxidant capacity of the blood. This aspect of nanoparticles in physiological fluids is further studied vastly by many investigators considering different nanoparticles. The combined heat and mass transfer phenomenon was investigated by Ali et al. [19] in the peristaltic motion of a nanofluid in a symmetric channel. Tripathi and Beg [20] discussed the use of the peristaltic motion of water-based nanofluid in the drug delivery process. A similar application was also reported by Abbas et al. [21] in a water-based nanofluid flow through a nonuniform microchannel. Some recent investigations in the field of nanofluid transport in different channels are mentioned in [22,23,24]. 

In various biological processes involving chemical reactions, another remarkable phenomenon is the deterioration in free energy. For instance, the metabolic system in living creatures produces chemical reactions to trigger the free energy which consequently results in the production of a considerable extent of entropy. Some antientropic actions include the flow of various materials, such as urination, sweat, blood stream, muscle spasms, and biosynthesis. Owing to these important applications of thermodynamics in biological systems, some investigations ([25,26]) have been conducted in this field. A remarkable contribution to entropy generation and its minimization were contributed by Bejan [27]. Furthermore, Bejan [28] studied entropy production in four separate modes of heat transfer. In the biological regime, Saleem and Munawar [29] analyzed the entropy generation trend in an inclined channel containing ciliated structures filled with non-Newtonian hyperbolic tangent fluid. An interesting contribution divulging the thermal analysis of Cu-water nano-liquid in a tube was given by Akbar and Butt [30]. Recently, a bio-magnetic liquid transport considering heat transfer aspect was studied by Saleem and Munawar [31]. 

In microchannels, at low shear rates many physiological fluids behave like non-Newtonian fluids due to their shear-thinning and elastic characteristics. Due to their rheological characteristics, some physiological fluids are modeled as Carreau fluid. In a comparison study, Johnson et al. [32] computed wall shear stress for various non-Newtonian fluid models and compared it with the experimental data, and found that Carreau fluid is the best fluid model for blood flow. Therefore, in the present study, we consider Carreau fluid as the base fluid.

A glance over the past literature reveals that the entropy problem in ciliated channels must be studied further under various physical assumptions, such as, under electroosmosis, thermal radiation, Joule heating, etc. The primary objective of this study is to explore the entropy generation aspects of thermally radiated nano-liquid in an electroosmotic pump with its surface lined with a cilia mat. Due to rheological characteristics, the Carreau fluid is considered as blood combined evenly with a 1−6% volume fraction of Copper nano-sized particles. The collective impact of Joule heating and radiative heat transfer has been considered while formulating the entropy expression. The problem has been transformed in the moving frame under the practical assumptions of long-wavelength approximation and the Debye-Hückel linearization. Roseland approximation has been used to linearize the thermal radiation term while the Debye-Hückel approximation has been applied to linearize the Poisson-Boltzmann equation. The simplified governing system has been solved numerically with the aid of the shooting technique. The results have been discussed with the aid of several graphs. The expression for pressure-rise per metachronal wavelength is numerically calculated by integrating the pressure gradient.

## 2. Mathematical Modelling

**Figure 1:**
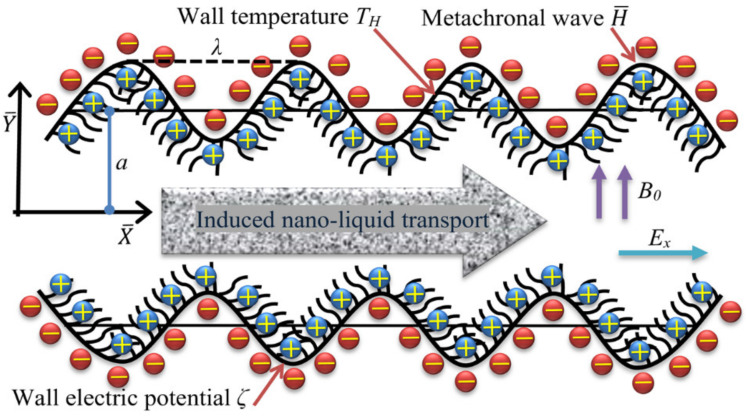
Schematic diagram of flow through the electroosmotic ciliated pump.

We consider a steady, two-dimensional flow of a Cu-blood nanofluid in a long channel contains hair-like structure at the wall, called cilia (see Figure 1). A synchronized metachronal traveling wave progressing with an angular velocity *c* along the flexible wall, thus, produces an electric current of intensity *E_x_* in the direction of flow. The surface temperature of the wall remains constant at a value *T_H_*. The effective and recovery strokes of the cilia field stimulate the rhythmic waves. The cartesian coordinate system is framed by taking the axis $\bar{X}$ along the wave transmission and the $\bar{Y}$-axis in the upright direction. The cilia geometry is expressed by the following wave function [33,34]:

(1) $$\bar{Y}=\bar{H}\left(\bar{X},t\right)=a+a\varepsilon \cos\left(\frac{2\pi }{\lambda }\left(\bar{X}-ct\right)\right).$$

Sleigh [35] proved in an experimental investigation that the cilia tip adopts an elliptic path, and are positioned with the channel wall at

(2) $$\bar{X}=G\left(\bar{X},t\right)=X_{0}+a\varepsilon \alpha \sin\left(\frac{2\pi }{\lambda }\left(\bar{X}-ct\right)\right),$$

where *ε* is the cilia length, α is the eccentricity of cilia’s elliptical motion, *a* symbolizes the mean width of channel, *λ* is the wavelength, *t* is the time, and *X*_0_ is the position of the fluid particle.

Differentiating Equations (1) and (2) with respect to *t* to obtain the velocity components at channel wall as

(3) $$U_{0}={\left(\frac{\partial \bar{X}}{\partial t}\right)}_{X_{0}}=\frac{-\left(\frac{2\pi }{\lambda }\right)ac\varepsilon \alpha \cos\left(\frac{2\pi }{\lambda }\left(\bar{X}-ct\right)\right)}{1-\left(\frac{2\pi }{\lambda }\right)a\varepsilon \alpha \cos\left(\frac{2\pi }{\lambda }\left(\bar{X}-ct\right)\right)},$$

(4) $$V_{0}={\left(\frac{\partial \bar{Y}}{\partial t}\right)}_{X_{0}}=\frac{-\left(\frac{2\pi }{\lambda }\right)ac\varepsilon \alpha \sin\left(\frac{2\pi }{\lambda }\left(\bar{X}-ct\right)\right)}{1-\left(\frac{2\pi }{\lambda }\right)a\varepsilon \alpha \sin\left(\frac{2\pi }{\lambda }\left(\bar{X}-ct\right)\right)}.$$

The flow is assumed to be under the influence of an external force **F** which is the sum of transverse magnetic field and an axially applied electric field and is given by

(5) $$F=\rho_{e}E+J\times B,$$

where **J** = *σ_nf_* (**V** × **B**) is the electric current density by Ohm’s law. Here **B** = (0, *B*_0_, 0) is external uniform magnetic field of strength *B*_0_ and **E** = (*E_x_*, 0, 0) represents intensity of axially applied electric field. Thus, the governing equations under the aforementioned suppositions in a stationary frame of reference are given by

(6) $$\frac{\partial \bar{U}}{\partial \bar{X}}+\frac{\partial \bar{V}}{\partial \bar{Y}}=0,$$

(7) $$\rho_{nf}\left(\frac{\partial \bar{U}}{\partial t}+\bar{U}\frac{\partial \bar{U}}{\partial \bar{X}}+\bar{V}\frac{\partial \bar{U}}{\partial \bar{Y}}\right)=-\frac{\partial \bar{P}}{\partial \bar{X}}+\frac{\partial {\bar{S}}_{XX}}{\partial \bar{X}}+\frac{\partial {\bar{S}}_{XY}}{\partial \bar{Y}}-\sigma_{nf}B_{0}^{2}\bar{U}+\rho_{e}E_{x},$$

(8) $$\rho_{nf}\left(\frac{\partial \bar{V}}{\partial t}+\bar{U}\frac{\partial \bar{V}}{\partial \bar{X}}+\bar{V}\frac{\partial \bar{V}}{\partial \bar{Y}}\right)=-\frac{\partial \bar{P}}{\partial \bar{Y}}+\frac{\partial {\bar{S}}_{XY}}{\partial \bar{X}}+\frac{\partial {\bar{S}}_{YY}}{\partial \bar{Y}},$$

(9) $$\begin{array}{l} {\left(\rho C_{P}\right)}_{nf}\left(\frac{\partial \bar{T}}{\partial t}+\bar{U}\frac{\partial \bar{T}}{\partial \bar{X}}+\bar{V}\frac{\partial \bar{T}}{\partial \bar{Y}}\right)\\ \;=k_{nf}\left(\frac{\partial^{2}\bar{T}}{\partial {\bar{X}}^{2}}+\frac{\partial^{2}\bar{T}}{\partial {\bar{Y}}^{2}}\right)+{\bar{S}}_{XX}\frac{\partial \bar{U}}{\partial \bar{X}}+{\bar{S}}_{XY}\left(\frac{\partial \bar{U}}{\partial \bar{Y}}+\frac{\partial \bar{V}}{\partial \bar{X}}\right)+{\bar{S}}_{YY}\frac{\partial \bar{V}}{\partial \bar{Y}}-\frac{\partial {\bar{q}}_{r}}{\partial \bar{Y}}\\ \;+\sigma_{nf}\left(E_{x}^{2}+B_{0}^{2}{\bar{U}}^{2}\right), \end{array}$$

Subject to the boundary conditions

(10) $$\left.\begin{array}{c} \bar{U}=U_{0},\;\;\;\;\bar{T}=T_{H}\;at\;\bar{Y}=\bar{H},\\ \frac{\partial \bar{U}}{\partial \bar{Y}}=0,\;\;\;\frac{\partial \bar{T}}{\partial \bar{Y}}=0\;at\;\bar{Y}=0, \end{array}\right\}$$

where $\bar{P}$ is the pressure, $\bar{T}$ the temperature field, *T_H_* the wall temperature, and $\left(\bar{U},\bar{V}\right)$ is the velocity vector. For Carreau fluid, the stress tensor is described as

(11) $$\bar{S}=-\left[\mu_{nf}{\left(1+{\left(\Gamma \overset{•}{\gamma }\right)}^{2}\right)}^{\frac{n-1}{2}}\right]\bar{\overset{•}{\gamma }}$$

with $\bar{\overset{•}{\gamma }}=\sqrt{\frac{1}{2}\sum_{i}\sum_{j}{\bar{\overset{•}{\gamma }}}_{\mathrm{ij}}{\bar{\overset{•}{\gamma }}}_{\mathrm{ji}}}=\sqrt{\frac{1}{2}\Pi }$.

The components of stress tensor in Equation (11) are given by

(12) $${\bar{S}}_{XX}=-2\mu_{nf}\left[1+\left(\frac{n-1}{2}\right)\Gamma^{2}{\overset{•}{\gamma }}^{2}\right]\frac{\partial \bar{U}}{\partial \bar{X}},$$

(13) $${\bar{S}}_{XY}=-\mu_{nf}\left[1+\left(\frac{n-1}{2}\right)\Gamma^{2}{\overset{•}{\gamma }}^{2}\right]\left(\frac{\partial \bar{U}}{\partial \bar{Y}}+\frac{\partial \bar{V}}{\partial \bar{X}}\right),$$

(14) $${\bar{S}}_{YY}=-\mu_{nf}\left[1+\left(\frac{n-1}{2}\right)\Gamma^{2}{\overset{•}{\gamma }}^{2}\right]\left(\frac{\partial \bar{V}}{\partial \bar{Y}}\right),$$

where *μ_nf_* is the effective viscosity, *Γ* is the fluid time relaxation parameter, *n* the power-law index and **Π** represents the second invariant strain tensor. The Carreau model given in Equation (11) exhibits the Newtonian fluid model at *n* = 1 and/or *Γ* = 0.

The default volume fraction of Cu nanoparticles assumed in the current study is 1–6% of the base fluid (Carreau fluid). The mathematical equations representing the attributes of Cu-blood nanofluid are listed as [36,37]:

(15) $$\rho_{nf}=\rho_{f}\left[\left(1-\phi \right)+\phi \left(\frac{\rho_{np}}{\rho_{f}}\right)\right],$$

(16) $$\mu_{nf}=\frac{\mu_{f}}{\sqrt{{\left(1-\phi \right)}^{5}}},$$

(17) $${\left(\rho C_{P}\right)}_{nf}=\left(1-\phi \right){\left(\rho C_{P}\right)}_{f}+\phi {\left(\rho C_{P}\right)}_{np},$$

(18) $$\sigma_{nf}=\sigma_{f}+\frac{3\left(\frac{\sigma_{s}}{\sigma_{f}}-1\right)\phi \sigma_{f}}{\left(\frac{\sigma_{s}}{\sigma_{f}}+2\right)-\left(\frac{\sigma_{s}}{\sigma_{f}}-1\right)\phi },$$

(19) $$\frac{k_{nf}}{k_{f}}=\frac{k_{np}+\left(S-1\right)k_{f}-\left(S-1\right)\phi \left(k_{f}-k_{np}\right)}{k_{np}+\left(S-1\right)k_{f}+\phi \left(k_{f}-k_{np}\right)},$$

where *ρ_f_*, *μ_f_*, (*ρC_P_*)*_f_*, *σ_f_*, *k_f_,* and *ϕ* are the density, viscosity, specific heat capacity, electrical conductivity, thermal conductivity, and the total volume fraction of solid nano particles, respectively, for the base fluid. Whereas, the subscripts “*np*” assign to these quantities corresponds to nanofluid characteristics. The numerical values of these characteristics are mentioned in Table 1. The parameter *S* represents the nanoparticles shape [38,39], such as, a value of *S* = 5.7 corresponds to lens-shaped nanoparticles and *S* = 4.7 represents cylindrical-shaped nanoparticles. In this study, *S* is considered to be lens-shaped (=5.7).

The radiation heat transfer is one of the heat transfer modes present in thick media flows. The radiative heat flux in the $\;\bar{X}$-direction is assumed to be negligible as compared to the $\;\bar{Y}$-direction. For intense absorption and a system in thermal equilibrium, Rosseland’s approximation [41] suggests that the radiative heat flux ${\bar{q}}_{r}$ is approximated by:

(20) $${\bar{q}}_{r}=-\frac{4\sigma^{*}}{3K^{*\;}}\frac{\partial {\bar{T}}^{4}}{\partial \bar{Y}},$$

where σ* is the Stefan-Boltzmann constant and K* the Rosseland mean spectral absorption coefficient. The fractional variation in temperature is considered to be adequately small in a distance of one mean free path. The first two terms of Taylor’s series of $\bar{T}$^4^ about the temperature difference are $\bar{T}$^4^ ≅ 4(*T*_1_−*T*_0_)^3^$\bar{T}$ − 3(*T*_1_ −*T*_0_)^4^. Thus, Equation (1) takes the form

(21) $${\bar{q}}_{r}=-\frac{16\sigma^{*}{\left(T_{1}-T_{0}\right)}^{3}}{3K^{*\;}}\frac{\partial \bar{T}}{\partial \bar{Y}}.$$

Transforming the variables from fixed frame to wave frame by utilizing the following conversions:

(22) $$\bar{x}=\bar{X}-ct,\;\;\;\bar{y}=\bar{Y},\;\;\;\bar{u}=\bar{U}-c,\;\;\;\bar{v}=\bar{V},\;\;\;\bar{p}\left(x,y\right)=\bar{P}\left(\bar{X},\bar{Y},t\right).$$

The distribution of electric potential $\bar{\Phi }$ is modeled by the Poisson-Boltzmann equation as [42]:

(23) $$\frac{\partial^{2}\bar{\Phi }}{\partial {\bar{X}}^{2}}+\frac{\partial^{2}\bar{\Phi }}{\partial {\bar{Y}}^{2}}=-\frac{\rho_{e}}{\epsilon \epsilon_{0}},$$

where *ϵ*_0_ is the permittivity of free space and *ϵ* is the medium permittivity. The parameter *ρ_e_* is the net charge density which is a function of $\bar{\Phi }$. In the case of binary fluid comprising of cation and anion, it can be expressed as:

(24) $$\rho_{e}=ez\left({\bar{n}}_{+}-{\bar{n}}_{-}\right),$$

where the positive and negative charges in bulk concentration are defined as

(25) $${\bar{n}}_{+}\left(=n_{0}e^{-\frac{ze\bar{\Phi }}{k_{b}T_{ave}}}\right)\;\mathrm{and}\;{\bar{n}}_{-}\left(=n_{0}e^{\frac{ze\bar{\Phi }}{k_{b}T_{ave}}}\right),$$

with *z* is the valence of type-i ions, *e* is the electric charge of a proton, *k_b_* is the Boltzmann constant, *n_0_* is the bulk ionic concentration. The concentration of nanofluid in Equations (15−19) is homogeneous, thus, there does not exist any concentration gradient in the fluid and the flow Peclet number is adequately insignificant. Such an assumption validates the distribution of ionic concentration.

For symmetric electrolytes, the net charge density can simply be computed as:

(26) $$\rho_{e}=2n_{0}ze\sin h\left(\frac{ze\bar{\Phi }}{k_{b}T_{ave}}\right),$$

To obtain an analytic solution of Equation (23), it is common to perform one further simplification and linearize the sine hyperbolic function to obtain the Debye-Hückel approximation [43]. Since the wall zeta potential is sufficiently small (≤25 mV), one obtains

(27) $$\sin h\left(\frac{ze\bar{\Phi }}{k_{b}T_{ave}}\right)≅\frac{ze\bar{\Phi }}{k_{b}T_{ave}},$$

Utilizing Equations (7) and (8), Equation (4) transforms to

(28) $$\frac{\partial^{2}\bar{\Phi }}{\partial {\bar{X}}^{2}}+\frac{\partial^{2}\bar{\Phi }}{\partial {\bar{Y}}^{2}}=\frac{2n_{0}z^{2}e^{2}}{k_{b}T_{ave}\epsilon \epsilon_{ο}}\bar{\Phi }$$

Introducing the following dimensionless quantities

(29) x=x¯λ, y=y¯a, Φ=Φ¯ζ, u=u¯c, v=λv¯ac, H=H¯a, t=ct¯a, p=p¯a2μfcλ,θ=T−T0TH−T0,β=aλ, Re=ρfacμf, Ha=σfμfB0a, Pr=μfCPfkf, Ec=c2CPfTH−T0,Rn=16σ*ΔT¯33μfCPfkf*, UHS=−Exϵϵοζcμf,We=Γca, Sp=σfEx2a2ΔT¯kf ,

where *x*, *y* are new independent variables, *u*, *v* are the dependent variables, *p* is the pressure, *θ* is the dimensionless temperature, *β* is the wave number, Ha is the Hartmann number, Pr is the Prandtl number, Ec is the Eckert number, *R_n_* is the thermal radiation number, *U_HS_* the Helmholtz-Smoluchowski velocity, We is the Weissenberg number for Carreau fluid, and *S_p_* is the term signifying Joule heating.

The velocity components in the stream function form can be written as *u* = ∂*Ψ*/∂*y* and *v* = –∂*Ψ*/∂*x*. Normalizing Equations (6−10) in light of Equations (22) and (29), and afterword employing the long wavelength and small Reynolds number approximation, the inertia effects become negligible [42] and the following set of equations are obtained:

(30) $$\frac{\partial p}{\partial x}=L_{1}\frac{\partial^{3}\Psi }{\partial y^{3}}+\frac{n-1}{2}L_{1}{\mathrm{We}}^{2}\frac{\partial }{\partial y}\left[{\left(\frac{\partial^{2}\Psi }{\partial y^{2}}\right)}^{3}\right]-L_{2}{\mathrm{Ha}}^{2}\frac{\partial \Psi }{\partial y}+U_{HS}\frac{\partial^{2}\Phi }{\partial y^{2}},$$

(31) $$\frac{\partial p}{\partial y}=0,$$

(32) $$\left(\frac{L_{3}}{\mathrm{Pr}}+R_{n}\right)\frac{\partial^{2}\theta }{\partial y^{2}}+\mathrm{Ec}L_{1}\left[{\left(\frac{\partial^{2}\Psi }{\partial y^{2}}\right)}^{2}+\frac{n-1}{2}{\mathrm{We}}^{2}{\left(\frac{\partial^{2}\Psi }{\partial y^{2}}\right)}^{4}\right]+\frac{L_{2}S_{p}}{\mathrm{Pr}}+L_{2}{\mathrm{EcHa}}^{2}{\left(\frac{\partial \Psi }{\partial y}\right)}^{2}=0.$$

Cross differentiation of Equations (30) and (31) leads to the following equation:

(33) $$L_{1}\frac{\partial^{4}\Psi }{\partial y^{4}}+\frac{\left(n-1\right)L_{1}}{2}{\mathrm{We}}^{2}\frac{\partial^{2}}{\partial y^{2}}\left[{\left(\frac{\partial^{2}\Psi }{\partial y^{2}}\right)}^{3}\right]-L_{2}{\mathrm{Ha}}^{2}\left(\frac{\partial^{2}\Psi }{\partial y^{2}}\right)+U_{HS}\frac{\partial^{3}\Phi }{\partial y^{3}}=0,$$

and the linearized Poisson-Boltzmann equation simplifies to

(34) $$\frac{\partial^{2}\Phi }{\partial y^{2}}=K^{2}\Phi ,$$

where

$L_{1}=\frac{1}{\sqrt{{\left(1-\phi \right)}^{5}}}$,
$L_{2}=\frac{\left(\frac{\sigma_{s}}{\sigma_{f}}+2\right)-\left(\frac{\sigma_{s}}{\sigma_{f}}-1\right)\phi +3\left(\frac{\sigma_{s}}{\sigma_{f}}-1\right)\phi }{\left(\frac{\sigma_{s}}{\sigma_{f}}+2\right)-\left(\frac{\sigma_{s}}{\sigma_{f}}-1\right)\phi }$,
$L_{3}=\frac{k_{np}+\left(S-1\right)k_{f}-\left(S-1\right)\phi \left(k_{f}-k_{np}\right)}{k_{np}+\left(S-1\right)k_{f}+\phi \left(k_{f}-k_{np}\right)}$,
and $K=aze\sqrt{\frac{2n_{0}}{\epsilon \epsilon_{0}k_{b}T_{ave}}}$ is the Debye-Hückel parameter.

The subsequent nondimensional boundary conditions are

(35) $$\left.\begin{array}{c} \Psi =0,\;\;\;\;\frac{\partial^{2}\Psi }{\partial y^{2}}=0,\;\;\;\frac{\partial \Phi }{\partial y}=0,\;\;\;\frac{\partial \theta }{\partial y}=0\;\;\;\;\;\;\;at\;\;\;\;\;\;\;y=0,\\ \Psi =F,\;\frac{\partial \Psi }{\partial y}=-1-\frac{2\pi \alpha \varepsilon \beta \cos2\pi x}{1-2\pi \alpha \varepsilon \beta \cos2\pi x},\;\;\;\theta =\Phi =1,\;at\;\;\;y=H=1+\varepsilon \cos2\pi x, \end{array}\right\}$$

Integrating the pressure gradient to obtain the pressure-rise per wavelength

(36) $$\Delta P=\int_{0}^{1}\left(\frac{dp}{dx}\right)dx.$$

The mean flow rates for fixed frame (Q) and for the wave frames (F) are given by:

(37) $$F=\int_{0}^{H}\left(\frac{\partial \Psi }{\partial y}\right)dy,\;\;\;\;\;\;Q=F+1.$$

Equations (32)–(34) along with boundary conditions (35) form a set of linear boundary value problem BVP. Such BVP can easily be solved in the Mathematica software with the help of the built-in “shooting” technique provided by the utility package “NDSolve”. We use this routine which solves *n*th order BVP as a system of *n* first-order initial value problems to obtain the exact numerical solution.

## 3. Entropy Analysis

For second-law analysis, consider the radiation, convection, viscous dissipation, and the Joule heating effects as the primary source of entropy production. Thus, the second-law yields ([44,45,46,47]):

(38) $$S_{gen}^{\prime \prime \prime }=\frac{k_{nf}}{T_{0}^{2}}\left(1+\frac{16\sigma^{*}{\left(T_{1}-T_{0}\right)}^{3}}{3K^{*\;}}\right)\left[{\left(\frac{\partial \bar{T}}{\partial \bar{X}}\right)}^{2}+{\left(\frac{\partial \bar{T}}{\partial \bar{Y}}\right)}^{2}\right]+\frac{\mu_{nf}}{T_{0}}\left[{\bar{S}}_{XX}\frac{\partial \bar{U}}{\partial \bar{X}}+{\bar{S}}_{XY}\left(\frac{\partial \bar{U}}{\partial \bar{Y}}+\frac{\partial \bar{V}}{\partial \bar{X}}\right)+{\bar{S}}_{YY}\frac{\partial \bar{V}}{\partial \bar{Y}}\right]+\frac{\sigma_{nf}}{T_{0}}\left(B_{0}^{2}{\bar{U}}^{2}+E_{x}^{2}\right).$$

Normalizing Equation (38) by assuming long wavelength approximations, incorporating Equations (22) and (29), and dividing with characteristic entropy, the expression for total entropy generation number is

(39) $$N_{G}=\left(L_{3}+R_{n}\right){\left(\frac{\partial \theta }{\partial y}\right)}^{2}+L_{1}\frac{\mathrm{PrEc}}{\tau }\left[{\left(\frac{\partial^{2}\Psi }{\partial y^{2}}\right)}^{2}+\frac{n-1}{2}{\mathrm{We}}^{2}{\left(\frac{\partial^{2}\Psi }{\partial y^{2}}\right)}^{4}\right]+L_{2}\frac{{\mathrm{PrEcHa}}^{2}}{\tau }{\left(\frac{\partial \Psi }{\partial y}\right)}^{2}+\frac{L_{2}}{\tau }S_{p},$$

where *τ* = Δ*T*/*T*_0_ represents the dimensionless temperature difference (assumed to be 1).

The Bejan number Be is given by:

(40) $$\mathrm{Be}=\frac{1}{1+\Xi },$$

where $\Xi =\frac{L_{1}\frac{\mathrm{PrEc}}{\tau }\left[{\left(\frac{\partial^{2}\Psi }{\partial y^{2}}\right)}^{2}+\frac{n-1}{2}{\mathrm{We}}^{2}{\left(\frac{\partial^{2}\Psi }{\partial y^{2}}\right)}^{4}\right]+L_{2}\frac{\mathrm{Pr}{\mathrm{EcHa}}^{2}}{\tau }{\left(\frac{\partial \Psi }{\partial y}\right)}^{2}+\frac{L_{2}}{\tau }S_{p}}{L_{3}\left(1+R_{n}\right){\left(\frac{\partial \theta }{\partial y}\right)}^{2}}$ is the irreversibilities ratio.

## 4. Results and Discussion

In this section, we discuss the graphical results of the present numerical solution and provide the physical interpretation. Several graphs are plotted for dynamically or thermodynamically important characteristics against various parameters of interest in Figure 2, Figure 3, Figure 4, Figure 5, Figure 6, Figure 7, Figure 8, Figure 9, Figure 10, Figure 11, Figure 12, Figure 13, Figure 14, Figure 15, Figure 16, Figure 17, Figure 18, Figure 19, Figure 20, Figure 21, Figure 22, Figure 23, Figure 24, Figure 25, Figure 26 and Figure 27. The Eckert number is kept fixed at 0.05, the nanoparticles concentration *ϕ* is 0.06, and the wave number *β* is 0.1 while the rest of the parameters are varied within suitable ranges.

**Figure 2:**
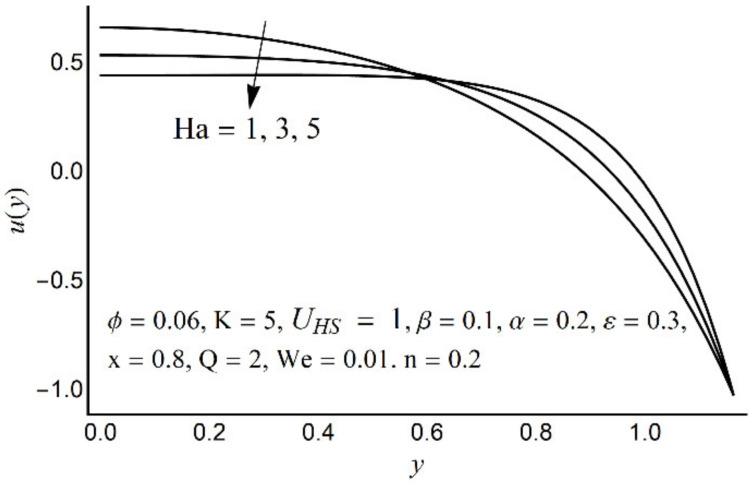
Axial velocity *u*(*y*) for various Ha.

**Figure 3:**
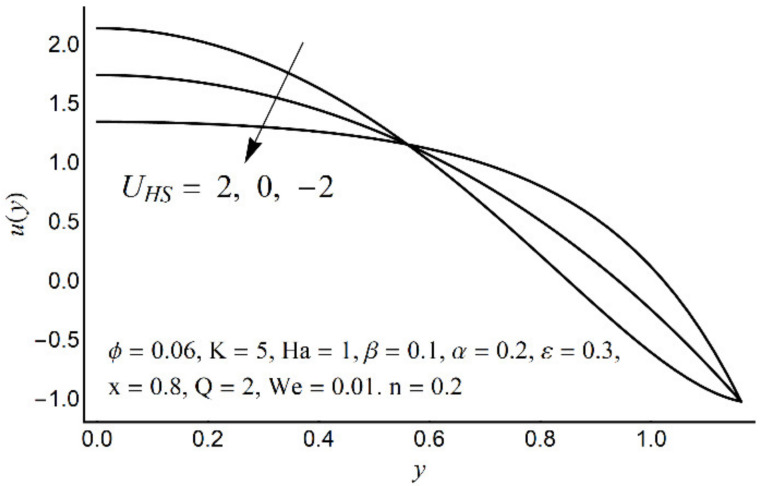
Axial velocity *u*(*y*) for various *U_HS_*.

**Figure 4:**
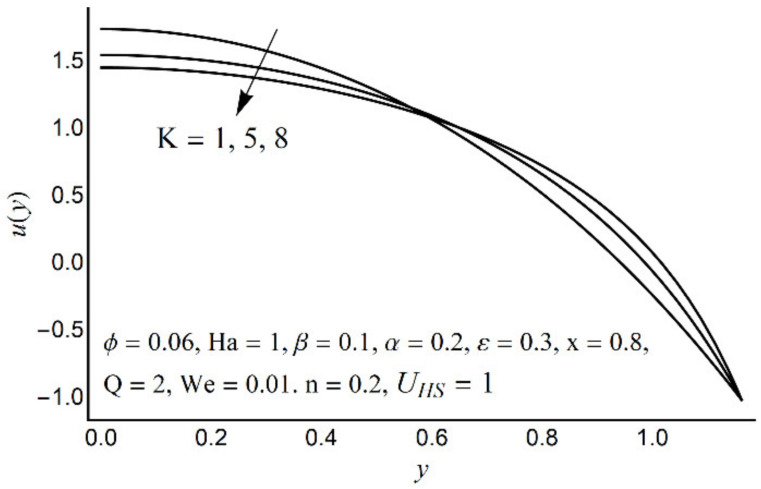
Axial velocity *u*(*y*) for various K.

**Figure 5:**
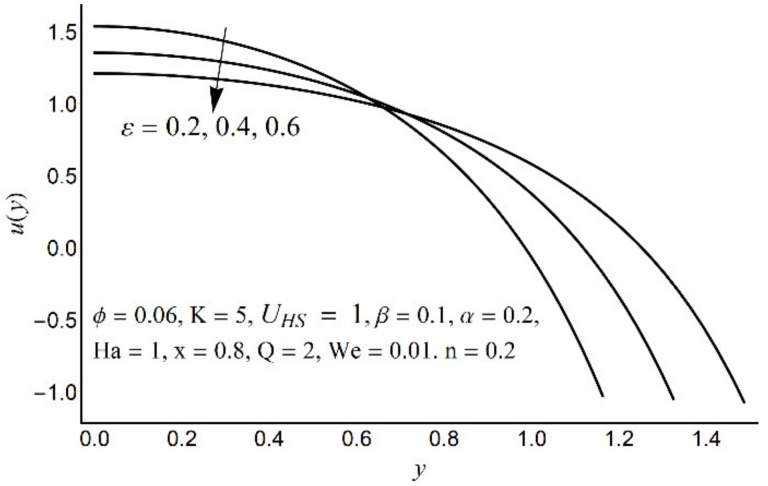
Axial velocity *u*(*y*) for various ε.

Figure 2, Figure 3, Figure 4 and Figure 5 describe the modifications in the axial velocity profile *u*(*y*) when different values of the Hartmann number (Ha), Helmholtz-Smoluchowski velocity parameter (*U_HS_*), Debye-Hückel parameter (K), and cilia length parameter (ε) are selected. Figure 2 reveals that an elevated value of Hartmann number (Ha) decelerates the fluid motion in the environs of the channel center and boosts near the channel boundary. Furthermore, this upsurge (in the Hartmann number) develops a more flattened pattern of velocity distribution close to the channel center. This declining conduct of Ha on fluid stream is due to the electromotive force which is linked to the magnetic force. The induced force possesses the ability to defy the fluid flow in the channel deep zone. But in a wavy channel, in order to preserve a constant flow rate, an utterly inverse state can be encountered near the channel boundaries. Figure 3 concluded that induction of Helmholtz-Smoluchowski velocity *U_HS_* in the flow direction enhances the fluid velocity. The same intensity of Helmholtz-Smoluchowski velocity, when applied in the reverse direction, produces a deceleration in fluid velocity especially in the center of the channel. The impact of the electroosmotic parameter (K) on the velocity field is shown in Figure 4. From the figure, it is observed that the large values of K reduce the fluid velocity at the center of the channel and hinder the velocity in the locality of the ciliated boundary. This conduct is quite expected since an increase in Debye thickness results in a strong electric double layer. Thus, the fluid velocity reduces at the channel center. However, near the wall of the channel, an entirely reverse behavior is noticed which signifies the momentum balance inside the channel. Figure 5 depicts that the prolonged cilia obstruct the flow in the deep channel region and exhibit trivial effects close to the ciliated wall. This behavior emerges as the cilium whip is directly proportional to its length. Therefore, a high value of the cilium length parameter proposes a considerable drop in fluid flow in the core channel region. 

**Figure 6:**
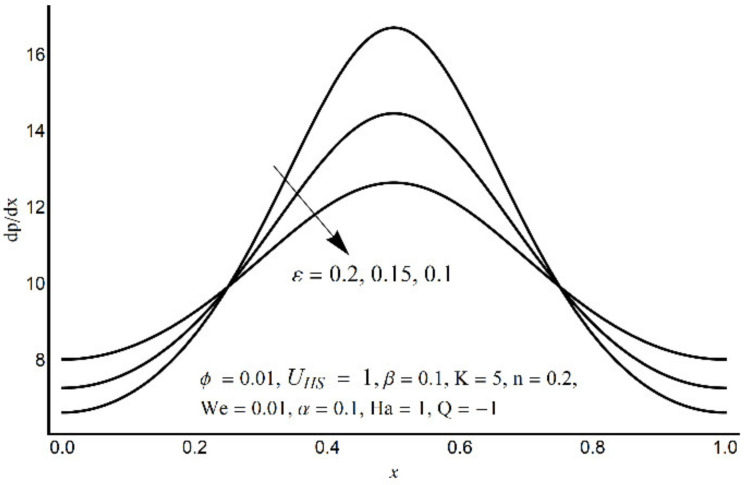
Pressure gradient at varying *ε*.

**Figure 7:**
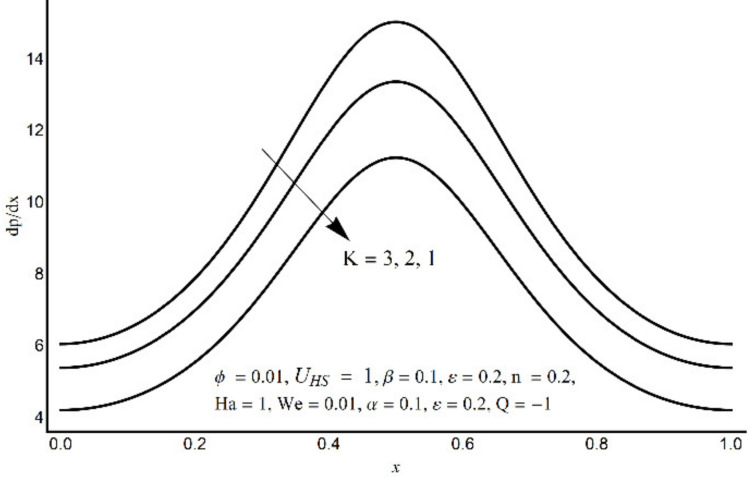
Pressure gradient at varying K.

**Figure 8:**
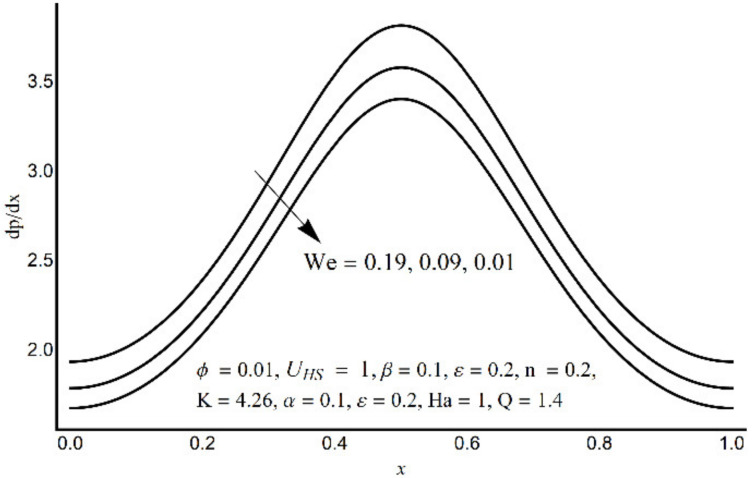
Pressure gradient at varying We.

**Figure 9:**
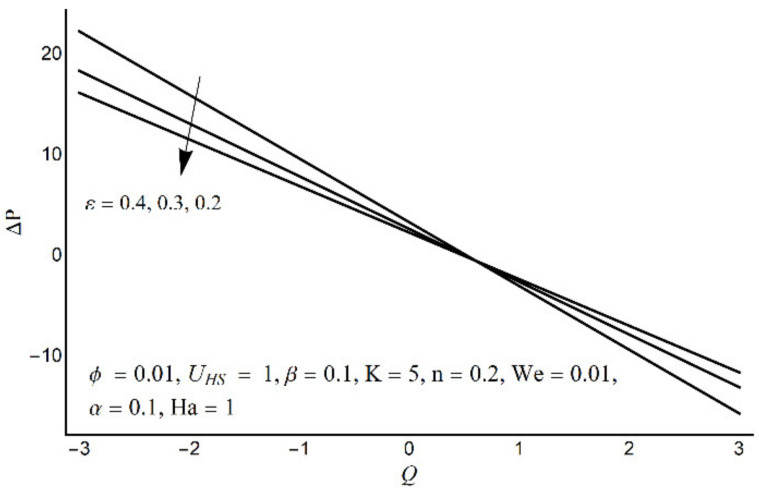
Pressure-rise per wavelength at varying *ε*.

**Figure 10:**
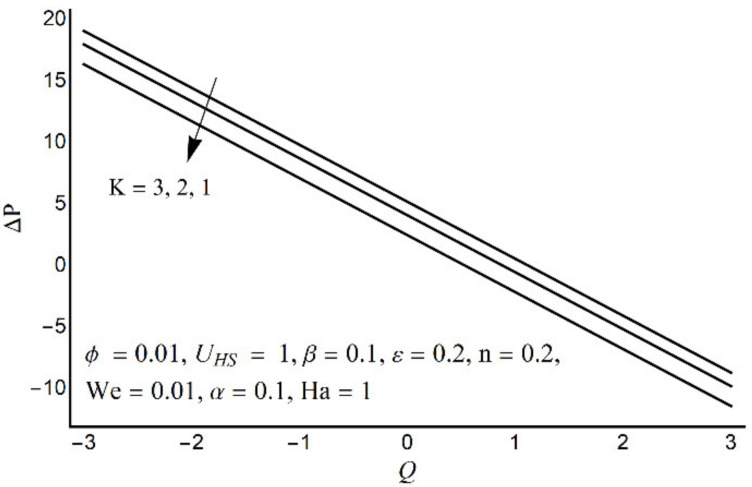
Effect of variation in K on pressure rise per wavelength.

Figure 6 depicts that the long appendages (coated channel surface) interact more acutely as compared to smaller ones. Therefore, an increase in cilia length parameter (*ε*) causes augmentation in the pressure gradient in the center of the channel, however, an inflected trend is noticed near the channel boundaries. Figure 7 and Figure 8 reveal that for large values of K (i.e., small Debye length), and the Weissenberg number We, the pressure gradient increases throughout the channel. This promising effect of these parameters is highly considered in the contracted cross-sections of the channel. Figure 9 indicates that in the pumping zone (∆P > 0), the ciliary motion is more influential than the peristaltic motion. However, this domination becomes weak in the free pumping area. In addition, in the augmented pumping zone (∆P < 0), the pressure rise per metachronal wavelength with a positive volume flow rate reflects a diminishing role of cilium length. From Figure 10, it is depicted that an enhancing behavior of electroosmosis parameter (K) on ∆P is retained throughout the pumping region.

**Figure 11:**
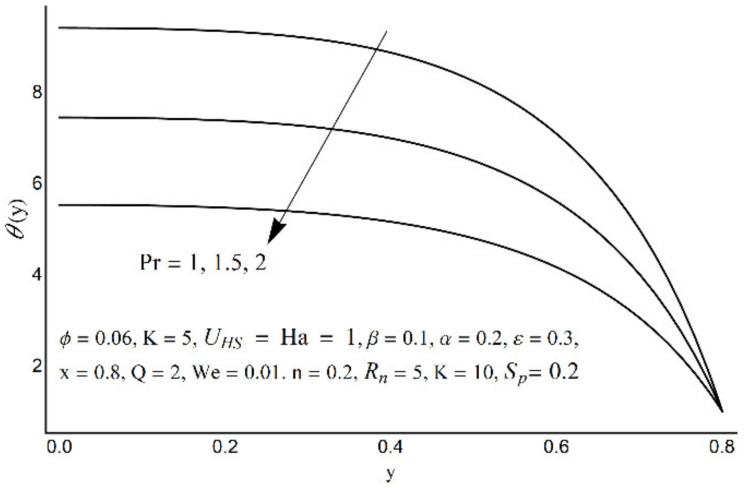
Temperature profile at various Pr.

**Figure 12:**
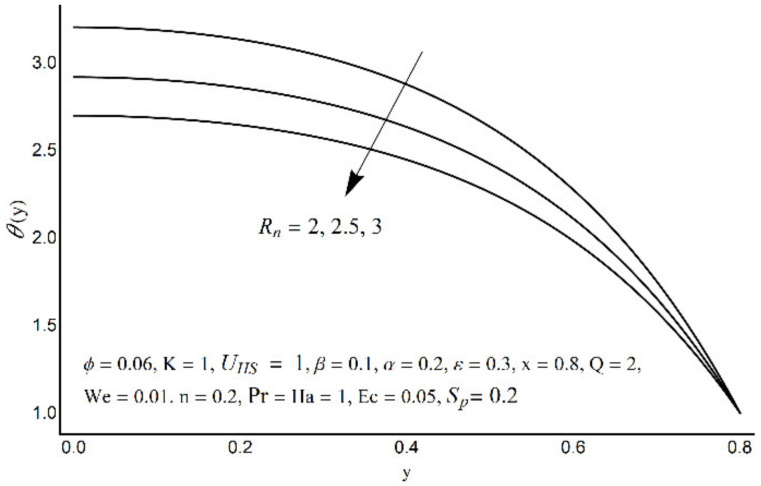
Temperature profile at various *R_n_*.

**Figure 13:**
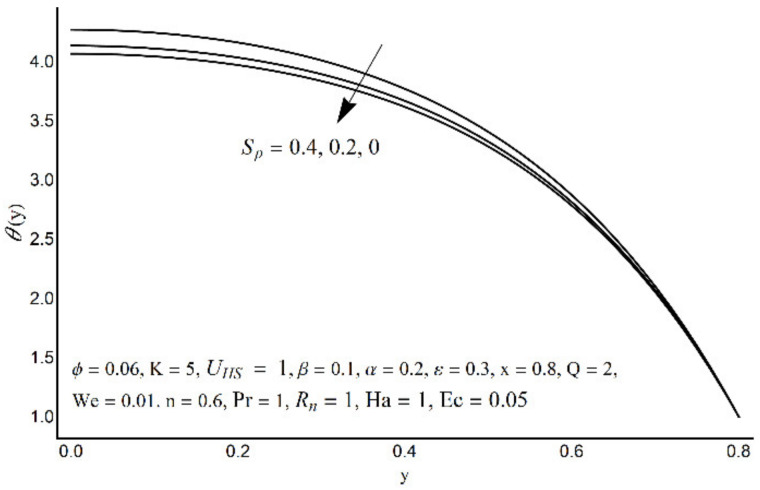
The temperature field for various values of *S_p_*.

**Figure 14:**
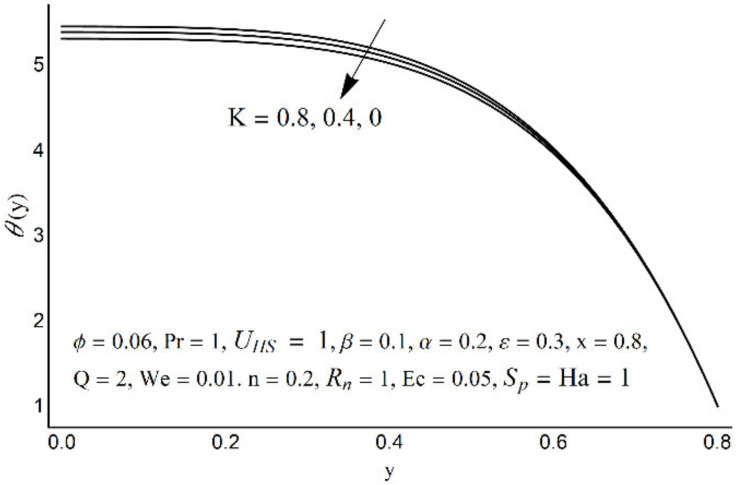
The temperature field for various values of K.

Variations in nanofluid temperature (*θ*) for different values of the Prandtl number (Pr), thermal radiation (*R_n_*), Joule heating (*S_p_*), and double electric layer (K) parameters are stated in Figure 11, Figure 12, Figure 13 and Figure 14. It is observed that the nanofluid temperature is enhanced when the Prandtl number rises (Figure 11). This rise is more considerable as one moves close to the channel center. A substantial drop in nanofluid temperature for large values of *R_n_* is reported in Figure 12. The Joule heating is known as the impact of an electric current passing through a conductor (medium) and produces thermal energy. Figure 13 shows that with an increase in the Joule heating parameter *S_p_*, the nanofluid temperature increases. Figure 14 reflects a remarkable augmentation in temperature for high values of K (due to an inverse relationship with the Debye length). However, this association is more significant near the middle of channel.

**Figure 15:**
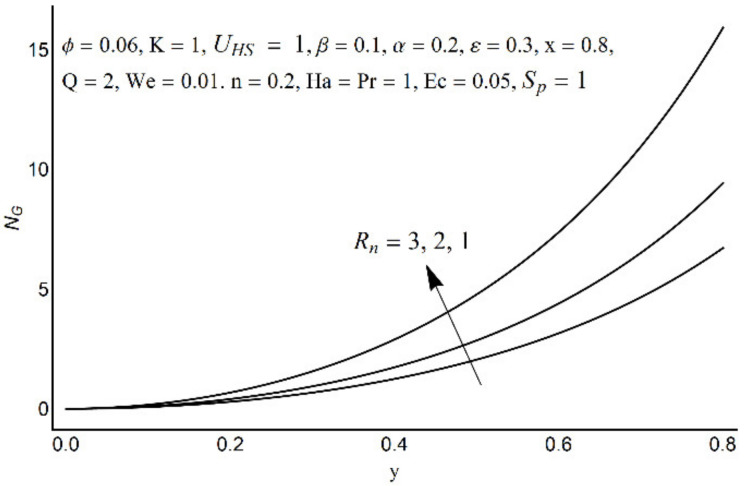
Entropy generation number for different values of *R_n_*.

**Figure 16:**
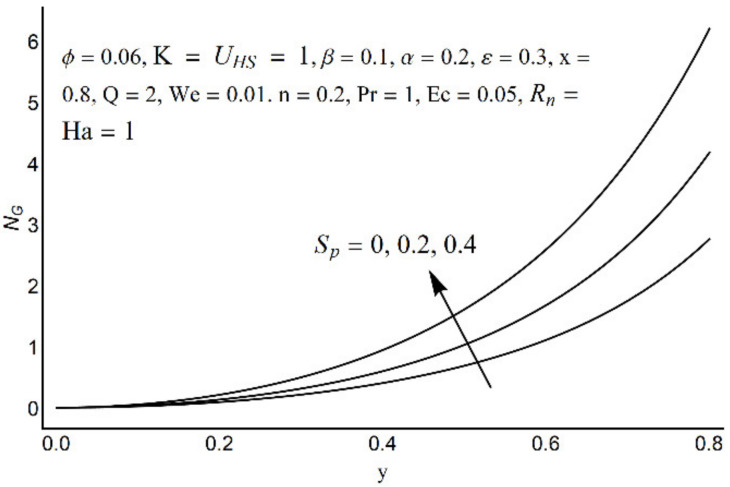
Entropy generation number for different values of *S_p_*.

**Figure 17:**
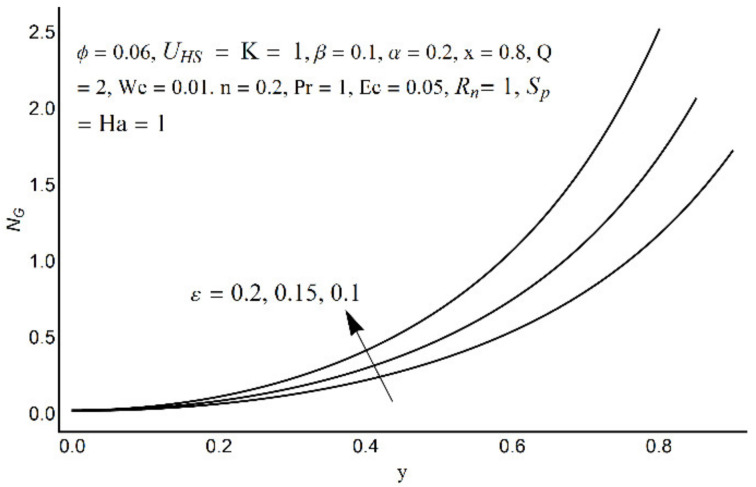
Entropy generation number for different values of *ε*.

**Figure 18:**
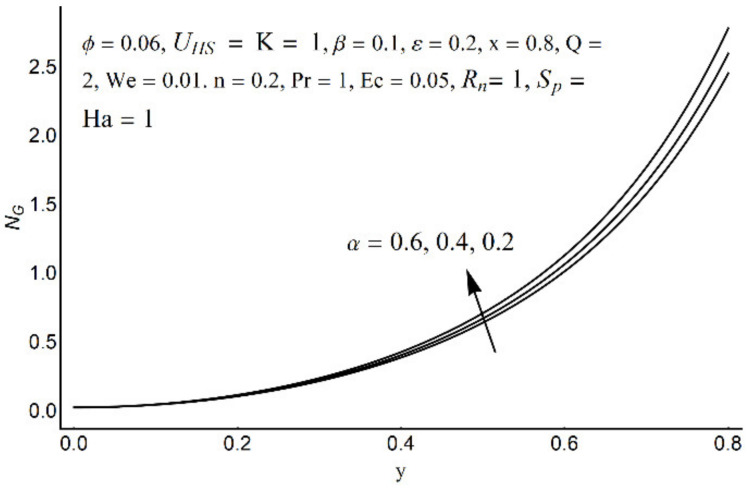
Entropy generation number for different values of *α*.

**Figure 19:**
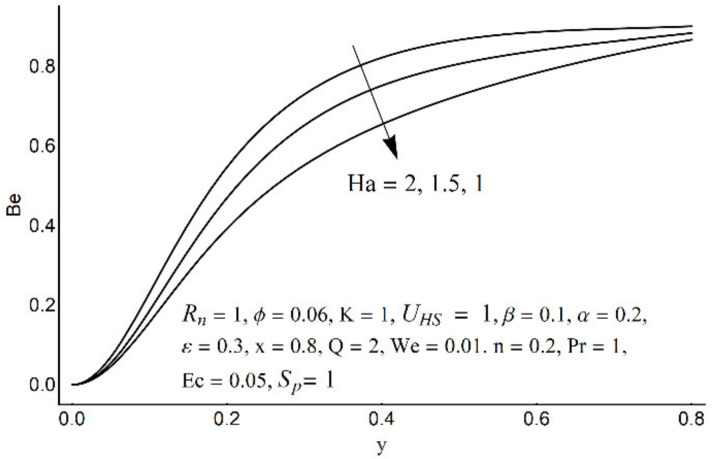
The Bejan number at various Ha.

**Figure 20:**
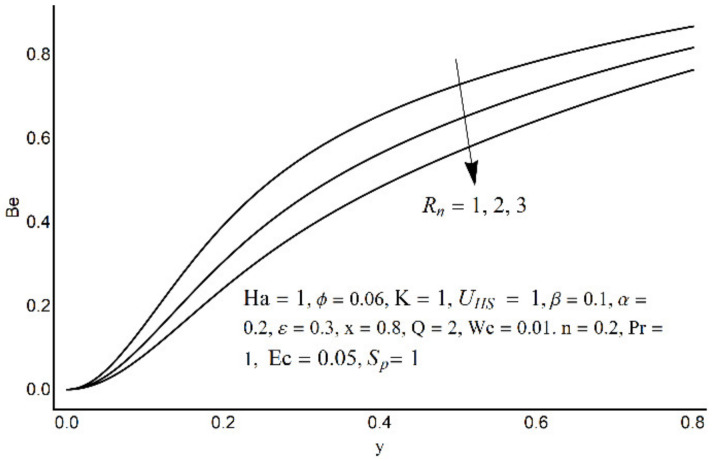
The Bejan number at various *R_n_*.

**Figure 21:**
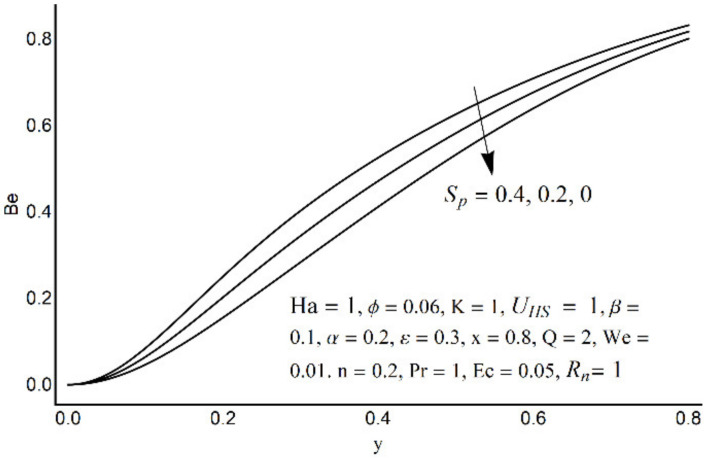
The Bejan number for different values of *S_p_*.

**Figure 22:**
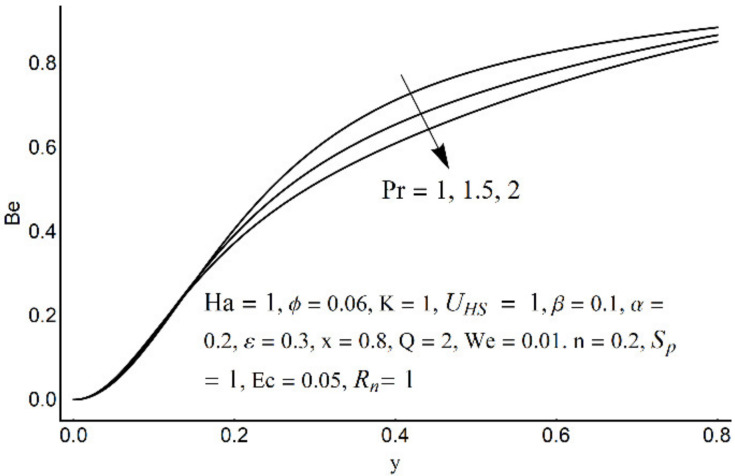
The Bejan number for different values of Pr.

**Figure 23:**
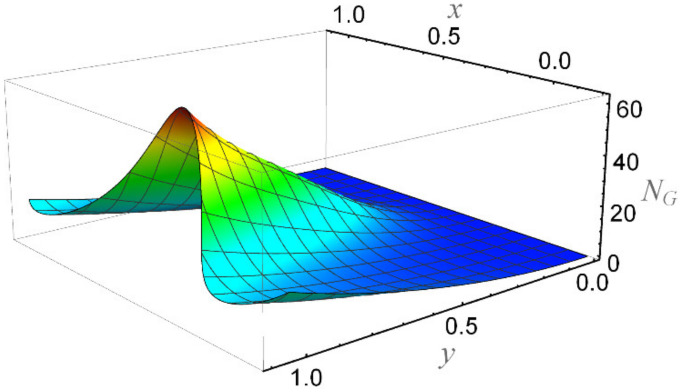
The entropy number when Br = 5, *ε* = 0.2, *α* = 0.3, We = 0.01, *β* = 0.1, K = 2, *R_n_* = 3, Ha = 1, *n* = 0.2, and Ec = 0.05.

**Figure 24:**
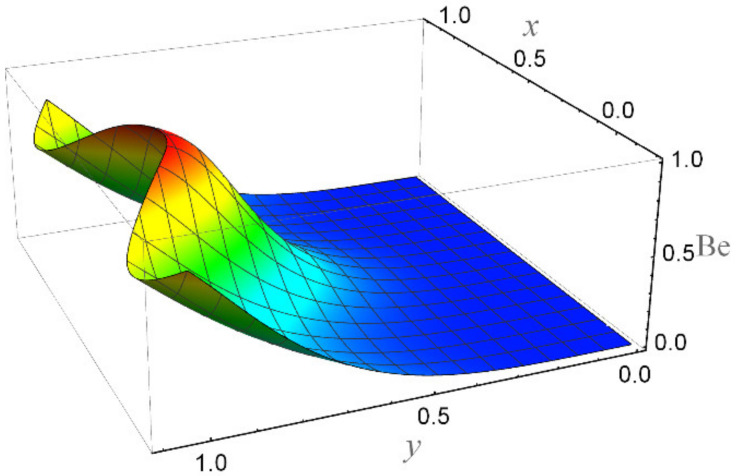
The Bejan number when Br = 5, *ε* = 0.2, *α* = 0.3, We = 0.01, *β* = 0.1, K = 2, *R_n_* = 3, Ha = 1, *n* = 0.2, and Ec = 0.05.

Figure 15 depicts that the overall entropy in the ciliated channel is diminished when high values of *R*_n_ are considered. However, an incredibly remarkable impact of this parameter on entropy generation can be seen in the surroundings of the channel wall. The Joule heating parameter *S*_p_ (Figure 16) reflects an absolutely opposite effect on entropy production when compared to Figure 15. From Figure 17 and Figure 18, it is concluded that the size and flexibility of the motile cilium perform an essential role in reducing the overall entropy production within the ciliated channel. More extended and elastic cilia have the ability to lessen the total entropy in the channel. Therefore, ciliary flows are found to be more antagonistic to entropy generation than peristaltic flows. Figure 19, Figure 20, Figure 21 and Figure 22 demonstrate the behavior of the Bejan number for the beating effects of fluid friction irreversibility and heat transfer irreversibility close to the channel center and wall, respectively. It is seen that the fluid friction and heat transfer irreversibilities rise for small values of heat radiation parameter *R*_n_ and for large values of the Hartmann number (Ha) and Joule heating (S_p_) parameters. Moreover, it is also noticed that the Prandtl number (Pr) has a lessening effect on heat transfer irreversibility but its impact on fluid friction irreversibility is insignificant. A three-dimensional glimpse of the Bejan number and total entropy generation number are plotted through Figure 23 and Figure 24. From Figure 23, it is noticed that the primacy of fluid friction irreversibility is associated with channel center. Whereas an ascendancy of heat transfer irreversibility is viewed close to the channel surface. Figure 24 reveals that the total entropy generation in the channel rises to its highest point in the contracted portion of the ciliated channel. Moreover, in the locality of the channel center, entropy production is nominal.

**Figure 25:**
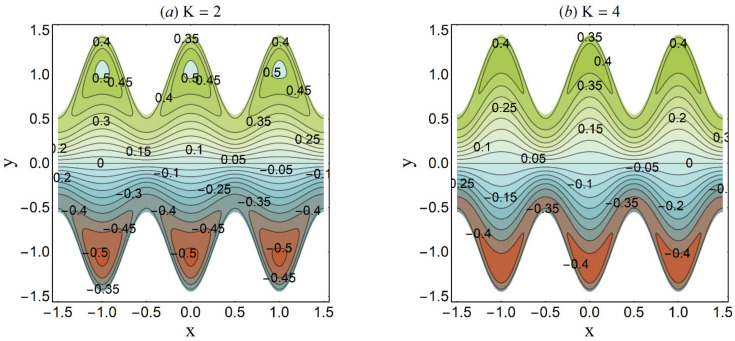
Streamlines for variation in K when *n* = 0.2 We = 0.05, *α* = 0.45, *ε* = 4, *β* = 0.1, *U_HS_* = 2, Ha = 1, *Q* = 0.4. (**a**) K = 2; (**b**) K = 4.

**Figure 26:**
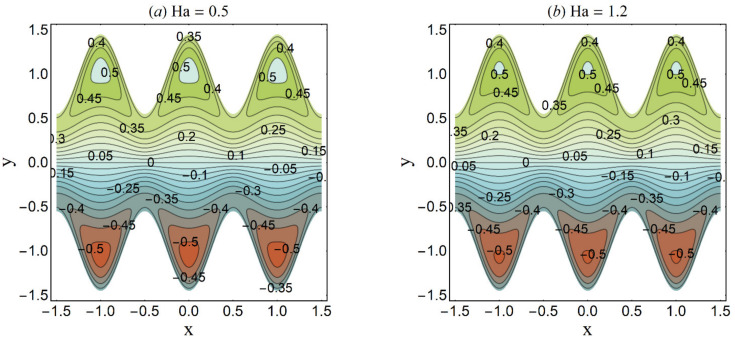
Streamlines for variation in Ha when *n* = 0.2 We = 0.05, *α* = 0.45, *ε* = 4, *β* = 0.1, *U_HS_* = 2, K = 2, *Q* = 0.4. (**a**) Ha = 0.5; (**b**) Ha = 1.2.

**Figure 27:**
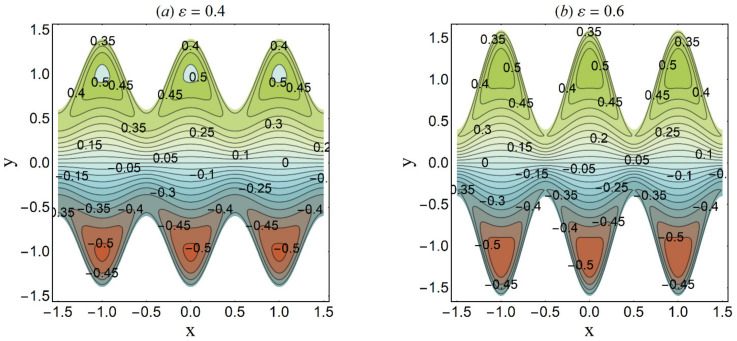
Streamlines for variation in *ε* when *n* = 0.2 We = 0.05, *α* = 0.45, Ha = 1, *β* = 0.1, *U_HS_* = 2, K = 2, *Q* = 0.4. (**a**) ε = 0.4; (**b**) ε = 0.6.

Trapping is an interesting trend noticed in the cilia-endorsed thrusting flows. It is defined as the development of an internally moving fluid mass surrounded by streamlines. Figure 25 established that the confined bolus shrinks in size when large values of the Debye-Hückel parameter (K) are considered. This suggests the decrease in flow rate at higher electroosmosis. Figure 26 depicts the same trend for the Hartmann number Ha on the streamlines. Since the main role of the magnetic force is to resist the fluid flow, thus, this trend meets the expectation. Figure 27 demonstrates that the bolus size expands as the cilia length ε increases. This shows a supportive role of the cilia structure in the channel to augment the fluid flow.

## 5. Conclusions

An entropy analysis is performed in a ciliated channel filled with nano-Carreau fluid under thermal radiation in the presence of an electric double layer EDL and magnetic field in the wave frame. The flow in the symmetric channel is stimulated by the metachronal waves and EDL. The numerical solution by the shooting method is presented. The following remarks conclude the present study:

1. Large values of magnetic and cilia length parameters support the fluid flow near the channel surface and are hindered near the core channel portion.
2. Electroosmosis parameter weakens the fluid stream near the channel wall and exhibits converse behavior near the channel center.
3. Large values of the cilia length parameter support the pressure gradient in the deep channel zone, whereas this behavior is sustained throughout the channel for the electroosmotic parameter and Weissenberg number.
4. In the pumping region, ciliary motion is more effective than peristaltic motion. But in the augmented pumping region, an opposite behavior is observed.
5. Electroosmosis parameter has an increasing effect on pressure rise and fluid temperature throughout the channel.
6. Fluid temperature escalates as Joule heating increases and thermal radiation decreases.
7. Total entropy inside the channel can be minimized by accomplishing an adequate thermally radiated fluid flow driven by the prolonged and more elastic cilia field.
8. Entropy in the channel is high for large values of the Joule heating parameter.
9. Entropy is observed to attain high values near the core channel part and stays small near the channel ciliated surface.
10. Trapping is enhanced as the cilia structure length grows and the electroosmotic parameter becomes smaller.

## Figures and Tables

**Table 1:** Thermophysical characteristics of base fluid and nano bits [40].

Physical Quantities	Base Fluid (Blood)	Solid Nanoparticles (Cu)
*ρ* (kg/m^3^)	1063	8933
*σ* (1/Ωm)	0.8	59.6 × 10^6^
*C_p_* (J/KgK)	3594	385
*k* (W/mK)	0.492	400

## Data Availability

Not applicable.
